# Synthesis of CaCO_3_ Nanobelts for Drug Delivery in Cancer Therapy

**DOI:** 10.1186/s11671-015-0948-6

**Published:** 2015-05-27

**Authors:** Dongmei Sun, Haibao Peng, Shilong Wang, Dazhang Zhu

**Affiliations:** School of Life Science and Technology, Tongji University, 1239 Siping Road, Shanghai, 200092 People’s Republic of China; State Key Laboratory of Molecular Engineering of Polymers and Department of Macromolecular Science, Fudan University, Shanghai, 200433 People’s Republic of China; Department of Chemistry, and Shanghai Key Lab of Chemical Assessment and Sustainability, Tongji University, 1239 Siping Road, Shanghai, 200092 People’s Republic of China

**Keywords:** CaCO_3_ nanobelts, Etoposide, Anti-cancer activity

## Abstract

Nanobelt carriers have demonstrated some advantages such as good biocompatibility, biodegradability, and strain-accommodating properties. We prepared an optimized nanobelt carrier formulation for drug (etoposide) as an oral delivery system and estimated the potential of calcium carbonate (CaCO_3_) nanobelts. The nanobelts were prepared by the method of binary solvent approach and were characterized by transmission electron microscope (TEM), scanning electron microscopy (SEM), and ultraviolet–visible (UV–vis) spectra. MTT (3-(4,5-dimethyl-2-thiazolyl)-2, 5-diphenyl-2H-tetrazolium bromide) assay test exhibited that etoposide-loaded calcium carbonate nanobelts (ECCNBs) showed a higher cell kill ratio against SGC-7901 cells compared with free drug. The apoptosis test and cell cycle test analysis revealed that etoposide entrapped in calcium carbonate nanobelts (CCNBs) could enhance the delivery efficiencies of drug and improved inhibition effect. The present findings demonstrated that ECCNBs might induce cell cycle arrest at G_2_/M phase and cell apoptosis in a p53-related manner. It can be foreseen that CCNBs are a promising drug carrier to store the anti-cancer drug for cancer therapy and drug delivery.

**Electronic supplementary material:**

The online version of this article (doi:10.1186/s11671-015-0948-6) contains supplementary material, which is available to authorized users.

## Background

Nowadays, cancer has become one of the main causes of death worldwide [1]. As cancer proliferates, many clinical cancer therapies have been developed, such as surgery, radiotherapy, and chemotherapy [2]. There existed some problems in chemotherapeutic drugs, such as the development of drug resistance, low bioavailability, and undesirable severe side effects [3, 4]. For example, etoposide (code designation VP-16-213, abbreviated VP16), a topoisomerase II inhibitor, is widely used as a chemotherapeutic agent currently in clinical trials for small cell lung cancer, lymphomas, and testicular cancers [5–8]. The formation of a complex of DNA-drug-enzyme can cause DNA double-stranded breaks to interfere with VP-16 topoisomerase II activity [9]. As we know, the entire VP16-induced apoptosis signaling pathways are not clear. But the DNA damage induced by VP16 has been shown that it can activate the activity of p53 [10, 11]. Therefore, it is significant to explore its signal path. Despite its extensive researches and developments, its clinical application was limited because of its side effect for normal tissue, extremely poor aqueous solubility, and it damages intestinal and hepatic metabolism [12, 13]. Therefore, some lab successfully solved the problem by developing the smart and innovative drug delivery systems in biomedical applications (such as superparamagnetic multistage delivery system [14], supramolecular microcapsules controlled release delivery system [15]).

Calcium carbonate (CaCO_3_) and its products have a wide application. CaCO_3_ is a typical biomaterial, which can be used in many products, such as cosmetics and toothpastes, and in the paper industry. Compared with other inorganic materials, calcium carbonate is exceedingly suitable because of its ideal properties of biodegradability and biocompatibility [16]. Furthermore, it has also been used as a smart carrier to deliver genes, enzymes, and drugs [17–22]. The property of pH sensitivity enables CaCO_3_ to be used for controlled degradability both in vitro and in vivo test [23].

Herein, we developed the novel CaCO_3_ nanobelts as the carrier for the drug delivery and cancer therapy. Meanwhile, the method is mild, environment-friendly, and easily performed, which is based on the binary solvent approach used by Peng [24]. We evaluate the drug-loading efficiency of etoposide-loaded calcium carbonate nanobelts (ECCNBs) and the drug release behaviors at different pH value. Otherwise, cellular experiments of MTT (3-(4,5-dimethyl-2-thiazolyl)-2, 5-diphenyl-2H-tetrazolium bromide) assay and cell cycle analysis were tested to evaluate the anti-cancer effect and biocompatibility of ECCNBs. Besides, the confocal laser scanning microscopy (CLSM) images were utilized to investigate the uptake ability of calcium carbonate nanobelts (CCNBs) by cancer cells. We also discussed the cellular uptake mechanism of the CCNBs. These results demonstrated that the nanobelts could be potentially applied as effective drug carrier for cancer treatment.

## Methods

### Materials

Etoposide (≥98 %) was a kind gift from the University of Science and Technology of China. Ethanol (analytical reagent (AR)), citric acid (AR), CaCl_2_ (AR), Na_2_CO_3_ (AR), and HCl (36–38 %) were purchased from Sinopharm Chemical Regent Co., Ltd. (Shanghai, China). Dimethyl sulfoxide (DMSO) and MTT formazan were purchased from Sigma Chemical Co. (St Louis, MO, USA). Dulbecco’s Modified Eagle Medium (DMEM), Roswell Park Memorial Institute (RPMI)-1640, fetal calf serum (FCS), streptomycin, trypsinase, and penicillin G were obtained from GIBCOBRL (Grand Island, New York, NY, USA). Deionized water was decarbonated when it was used in the experiments.

### Cell Lines and Culture Conditions

Human gastric carcinoma cells (SGC-7901) obtained from the Chinese Academy of Sciences Cells Bank, Shanghai, China, were routinely cultured in RPMI-1640 cell medium supplemented with 10 % FBS, 100 U/mL penicillin, and 100 mg/mL streptomycin, at 37 °C in 5 % CO_2_ and 95 % air atmosphere with >95 % humidity. All experiments were performed on cells in the logarithmic phase of growth.

### Synthesis of ECCNBs

ECCNBs were synthesized by mixing CaCl_2_, citric acid, and Na_2_CO_3_ aqueous solution, which also include etoposide and ethanol. Solution I: 10 mL CaCl_2_ (0.1 M) and 0.2 g etoposide were dissolved in 40 mL of the water and 20 mL of ethanol mixtures. Solution II: 10 mL of Na_2_CO_3_ (0.1 M) and 0.2 g citric acid were dissolved in 40 mL of the water and 20 mL of ethanol mixtures. Solution II was added dropwise to the Solution I with a vigorously stirred speed of 1000 rpm. After 72 h, we can obtain milky white precipitation. The precipitation was washed thrice with 40 mL of the water and 20 mL of ethanol mixtures first and then should be dried by vacuum freeze drier. The synthetic parameters of the blank carrier (CCNBs) were similar to the ECCNBs sample. The difference between CCNBs and ECCNBs is without the addition of VP16.

### Physical Characterization of Nanobelts

The obtained products were characterized by scanning electron microscopy (SEM, Hitachi S4800, Chiyoda-Ku, Japan) and transmission electron microscope (TEM). The morphological and spatial studies were carried out with field emission scanning electron microscopy (FESEM, JEOL 1230, Tokyo, Japan) at an accelerating voltage of 1–5 kV. The surface morphology of CCNBs was obtained by transmission electron microscope.

### Fourier Transform Infrared (FTIR) Spectral and Ultraviolet–visible (UV–vis) Spectral Study

FTIR measurement was recorded on a Bruker Vector 22 spectrophotometer (Madison, WI, USA) using the standard KBr disk method in the range of 4000 to 500 cm^−1^. For FTIR spectral study, CCNBs, free VP16, and ECCNBs were ground into power with KBr to get the pellets by applying a pressure of 300 kg/cm^2^. We measured the absorbance spectra of native etoposide (dispersed in ethanol), ECCNBs (dispersed in ethanol), and void CCNBs (dispersed in ethanol) through a Cary 50 UV-visible absorbance spectrophotometer (Varian, Victoria, Australia).

### Dispersity Study in RPMI-1640 Medium

Five milligrams etoposide was resuspended in 10 mL RPMI-1640 medium, which was supplemented with 1 % penicillin-streptomycin and 10 % fetal bovine serum solution in a glass bottle. The same quantity of etoposide was applied to ECCNBs for the dispersity study, based on the drug encapsulation efficiency.

### In Vitro Release Test

Ten milligrams of ECCNB sample was placed in a 15-mL tube. At the same time, 200 μL of 6 M HCl solution was added into it. Then the tube was filled with phosphate buffer solution (pH = 7.4) until it reached 10 mL. The concentration of VP16 can be monitored at 285 nm through a UV–vis spectrophotometer, when the ECCNBs sample was totally dissolved. The drug-loading capacity is work out as follows:

$$ \mathrm{drug}\hbox{-} \mathrm{loading}\kern0.5em \mathrm{capacity}\kern0.5em =\kern0.5em \frac{\mathrm{weight}\kern0.5em \mathrm{of}\kern0.5em \mathrm{etoposide}\kern0.5em \mathrm{in}\kern0.5em \mathrm{ECCNBs}}{\mathrm{weight}\kern0.5em \mathrm{of}\kern0.5em \mathrm{ECCNBs}}\times 100\% $$

The release test was researched in a system of 200 mL PBS at pH 4.5 and 7.4, respectively. Twenty-five milligrams ECCNBs was resuspended in a dialysis bag of 10 mL PBS. The release system was conducted in a bath reciprocal shaker with 100 rpm at 37 °C. Two milliliters liquid was extracted at desired time intervals. Another 2 mL of fresh PBS was added to the release system. The accumulated amount of etoposide released was measured at 285 nm by UV absorption.

### Cytotoxicity Assay

The cytotoxicity of CCNBs against the human embryonic kidney (HEK) 293T cells was tested by the MTT assay method. The 293T cells (1 × 10^4^ cells/well) were seeded on a 96-well polystyrene plate. One hundred microliters of DMEM (high glucose) medium supplemented with 1 % penicillin-streptomycin and 10 % fetal bovine serum solution was added to each well. After 24 h incubation, CCNBs were added to the wells with a concentration of 5, 10, 20, and 40 μg/mL in sequence. The 293T cells were incubated for another 24 or 48 h, respectively. The control experiment was added with pure culture medium without any treatment. Before mediums were removed, the 293T cells should be incubated with 20 μL of 5 mg/mL MTT for 4 h under light-blocking condition. Then 150 μL of DMSO was added into each well. The absorbance of 490 nm can be measured by ELX 800 UV reader. Cell viability can be work out by means of the following formula:

$$ \mathrm{Cell}\kern0.5em \mathrm{viability}\kern0.5em \left(\%\right)=\frac{{\mathrm{OD}}_{490}\left(\mathrm{test}\right)\hbox{-} {\mathrm{OD}}_{490}\left(\mathrm{blank}\right)}{{\mathrm{OD}}_{490}\left(\mathrm{control}\right)\hbox{-} {\mathrm{OD}}_{490}\left(\mathrm{blank}\right)}\times 100\% $$

### Inhibition Against SGC-7901 Cells

The anti-tumor effect of CCNBs, ECCNBs, and VP16 against SGC-7901 cells was conducted by MTT test. SGC-7901 cells (8 × 10^4^ cells/well) were seeded on a 96-well polystyrene plate. One hundred microliters of RPMI-1640 medium supplemented with 10 % fetal bovine serum and 1 % penicillin-streptomycin solution was added to each well. After 24 h incubation, ECCNBs were added to the wells with a concentration of 5, 10, 20, and 40 μg/mL in sequence. The SGC-7901 cells were incubated for another 24 or 48 h, respectively. The control experiment was added with pure culture medium without any treatment. Before mediums were removed, the SGC-7901 cells should incubate with 20 μL of 5 mg/mL MTT for 4 h under light-blocking condition. Then 150 μL of DMSO was added into each well. The absorbance of 490 nm can be measured by ELX 800 UV reader. The inhibition can be work out by means of the following formula:

$$ \mathrm{Inhibition}\kern0.5em \left(\%\right)=\left[1\hbox{-} \frac{{\mathrm{OD}}_{490}\left(\mathrm{test}\right)\hbox{-} {\mathrm{OD}}_{490}\mathrm{blank}}{{\mathrm{OD}}_{490}\left(\mathrm{control}\right)\hbox{-} {\mathrm{OD}}_{490}\mathrm{blank}}\right]\times 100\% $$

### Fluorescence-Activated Cell Sorter Analysis

Apoptosis assay was conducted with SGC-7901 cells by an Annexin V-Propidium Iodide (PI) detection kit (KeyGEN Biotech). SGC-7901 cells were suspended in RPMI-1640. Then, the cells were incubated for 24 h at 37 °C. Cells were pretreated with ECCNBs at the concentration of 30 μg/mL for another 24 h. According to the drug efficiency, the same quantity of etoposide was applied to the apoptosis analysis with another incubation of 24 h. At the prescribed time, the cells were harvested by trypsinization and washed with ice-cold PBS for 5 min. Then the binding buffer (140 mM NaCl, 10 mM HEPES, 2.5 mM CaCl_2_, pH 7.4) should be filled with PI and Annexin V directly. It should be incubated in the dark for another 15 min at 37 °C. Then the cells were monitored on a spectrophotometer of Beckton-Dickinson (Mountain View, CA, USA) for fluorescence-activated cell sorting (FACS) analysis.

### Confocal Laser Scanning Microscopy

Confocal laser scanning microscope (Leica, Wetzlar, Germany) equipped with an oil immersion objective (×60, Zeiss, Oberkochen, Germany) was used to obtained CLSM images of cellular internalization of VP16 and ECCNBs. Fluorescein isothiocyanate (FITC; 1.5 mg) and 7.67 mg ECCNBs dispersed in 10 mL PBS at 4 °C overnight avoid light. FITC can incorporate into ECCNBs by covalent attachment. The resultant suspension was centrifuged at 8000 rpm to remove the supernatant. Briefly, SGC-7901 cells with a density of 1.5 × 10^5^ cells per well were seeded in a 24-well plate at a seeding in 2 mL of growth medium. The cells should have an incubation time of 24 h at 37 °C. The attached cells should be treated with an equivalent dose of native etoposide of ECCNBs. After the prescribed time (1, 2, and 4 h), the cells were washed thrice (PBS, pH 7.4), collected by trypsinization, and centrifuged for 5 min at 1000 rpm. Fluorescence images were acquired at an excitation wavelength of 405 and 488 nm.

### Qualitative Cellular Uptake Study via Confocal Microscopy and Flow Cytometry

For qualitative cellular uptake tests, confocal microscopy and flow cytometry were both used. SGC-7901 cells (a seeding density of 1.5 × 10^5^ cells/well) were seeded in Bioptech tissue culture plates (Bioptechs Inc., Butler, PA), and 2.0 × 10^5^ cells/well on a 6-well polystyrene plate for confocal studies and flow cytometry studies, respectively. After 24 h incubation at 37 °C for attachment, the attached cells were then treated with a concentration (30 μg/mL) of native VP16 and ECCNBs. For confocal studies, the cells were incubated for 1, 2, and 4 h and then washed thrice with PBS (pH 7.4) to remove excessive ECCNBs or native etoposide. Fresh DMEM (high glucose) medium was added to the plates. Then the cells were viewed by a confocal laser scanning microscope. For flow cytometry studies, the cells were incubated for 4 h and then washed thrice with PBS (pH 7.4) to remove excess ECCNBs or native etoposide and trypsinized. After the collection of the cell, the PBS (pH 7.4) was used to wash it. Consequently, the cells were resuspended with 500 μL PBS (pH 7.4) and transferred into flow cell tubes. FITC fluorescence was analyzed by FACS Aria II (Beckton and Dickinson, Sanjose, CA), after excitation using a 488-nm argon laser. Fluorescence emission above 530 nm from 10,000 cells were collected, amplified, and scaled to generate single parameter histogram.

### Cell Cycle Analysis by Flow Cytometry

Flow cytometry was used to analyze the cell cycles. To analyze the cell cycles, we used a slightly different procedure. After an overnight plating in 6-well per well, cells were incubated with 30 μg/mL etoposide, CCNBs, and ECCNBs for 24 h. At time intervals, the coverslips with adherent SGC-7901 cells were harvested and washed with ice-cold PBS twice after 24 h of treatment. Cells were fixed in precooled 70 % ethanol (diluted with PBS) for 2 h and then washed with ice-cold PBS twice; after it was suspended with budding buffer, the cells were treated with RNaseA and stained with propidium iodide for 30 min at room temperature. Flow cytometer was used to analyze the stained cells.

### Western Blot Analysis

Casepase-7, casepase-8, casepase-9, and Cyt C (mitochondrial cytochrome C) antibodies were purchased from Cell Signaling Technology. The antibody of p53 was purchased from Santa Cruz Biotechnology. Briefly, the SGC-7901 cells were assessed by the western blot analysis. Then, the total protein was extracted by chemical methods. Protein content was determined by BCA protein assay reagent (KeyGen, Nanjing, China). Equal amount of the proteins (50 μg) of each sample was resolved on SDS-PAGE and then electro transferred to PVDF membrane (Millipore Corp.). Nonspecific binding was blocked by incubation with 5 % nonfat milk in Tris-buffered saline containing 0.1 % Tween-20 (TBST) for 1 h at room temperature. The blots were probed with respective primary antibodies (antibodies used were against p53, casepase-7, casepase-8, casepase-9, Cyt C, and β-actin in 1:1000 dilutions) at room temperature overnight and washed three times with TBST. The blots were then incubated with secondary antibody for 30 min and washed again three times with TBST, and signals were visualized by chemiluminescent ECL detection system.

### Statistical Analysis

Results were expressed as mean ± standard deviation. Statistical analyses were performed by using Student’s *t* test. Values of *P* <0.05 were considered statistically significant.

## Results and Discussion

**Fig. 1:**
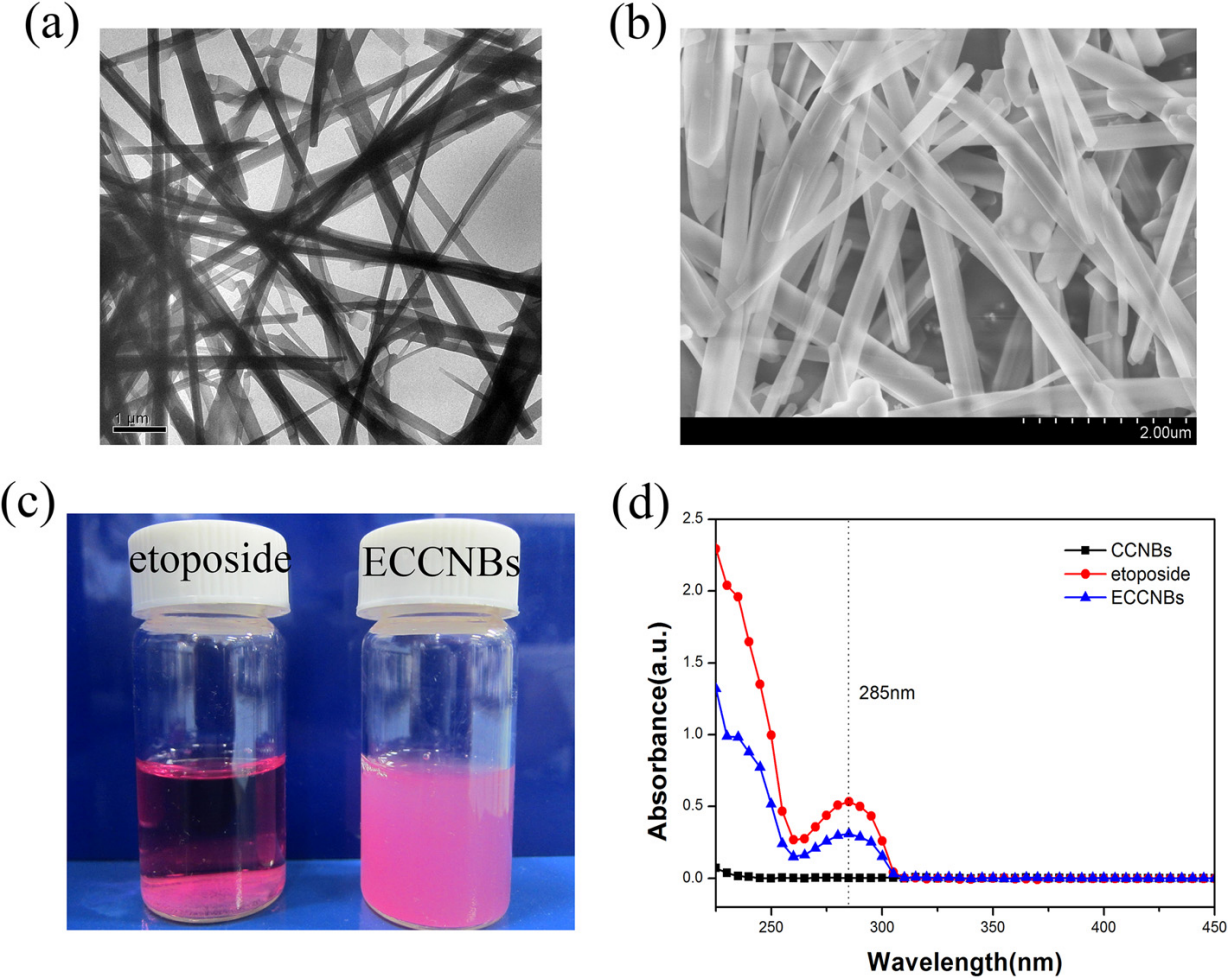
TEM (**a**) and SEM (**b**) image of CCNBs. **c** Sedimentation photographs of free etoposide and ECCNBs in RPMI-1640 medium. **d** UV–vis spectra for CCNBs, free etoposide, and ECCNBs

Nanobelts are a class of nanostructure, which are usually made from semiconducting metal oxides (such as CdSe, CdO, In_2_O_3_ or SnO_2_, selenides such as ZnO [25–27]). Nanobelts form ribbon-like structures with thicknesses of 10–30 nm, widths of 30–300 nm, and lengths in the millimeter range. They are structurally uniform single crystals with smooth surfaces and clean edges, possessing rectangular cross sections [28, 29]. The morphology and size of the CCNBs were characterized by the TEM and SEM photos (Fig. 1a, b). The particle size distribution of the nanoparticles was found to be relatively narrow.

The spectra of etoposide (Additional file 1: Figure S1c) show the following bands: 1056 cm^−1^ (C–O–C stretch), 1613 cm^−1^ (C=O stretch of carboxyl methyl), 1770 cm^−1^ (C=O stretch of ester bond), and 2923 cm^−1^ (C–H stretch), with the bands 1486 and 1404 cm^−1^ (C=C stretch) [30]. CaCO_3_ shows characteristic absorption peak centered at 875 cm^−1^ benefits its infrared absorption spectrum [31]. Additional file 1: Figure S1a, CCNBs display two strong absorption bands at 875 and 1426 cm^−1^, which are characteristic absorption bands of calcite. In the FTIR spectra, characteristic CO_3_^2−^ peak at 1417 cm^−1^ and C–O stretching at 1084 cm^−1^ are present. Compared with etoposide (c) and CCNBs (a), the spectra of ECCNBs (b) display the visible characteristic bands of CaCO_3_ and also show almost all characteristic vibration absorption bands of etoposide. The result of FTIR spectra indicates that CaCO_3_ nanobelts remained unchanged in the end.

The drug-loading capacity was calculated as 45 ± 3 %. To confirm the loading capacity and formulation of etoposide loaded in CCNBs, the photophysical property of etoposide was taken into consideration. Free etoposide, which dissolved in ethanolic solution, demonstrated its characteristic absorbance peak at 285 nm (Fig. 1d). The absorption spectrum of ECCNBs also showed an absorption band centered at 285 nm, which indicated the existence of etoposide in ECCNBs. The results of UV–vis spectra further demonstrated that the etoposide was successfully packed into the CCNBs.

Etoposide is a hydrophobic compound that is insoluble in aqueous solution. To confirm whether our formulation can increase etoposide’s dispersity, the same amount of native etoposide and ECCNBs were suspended in an equal volume of RPMI-1640 medium. We found that ECCNBs dissolved in a medium solution gave a well-dispersed status (Fig. 1c) in a medium solution. Therefore, the embedding of etoposide into CCNBs enhanced the dispersion of the drug in a medium solution.

**Fig. 2:**
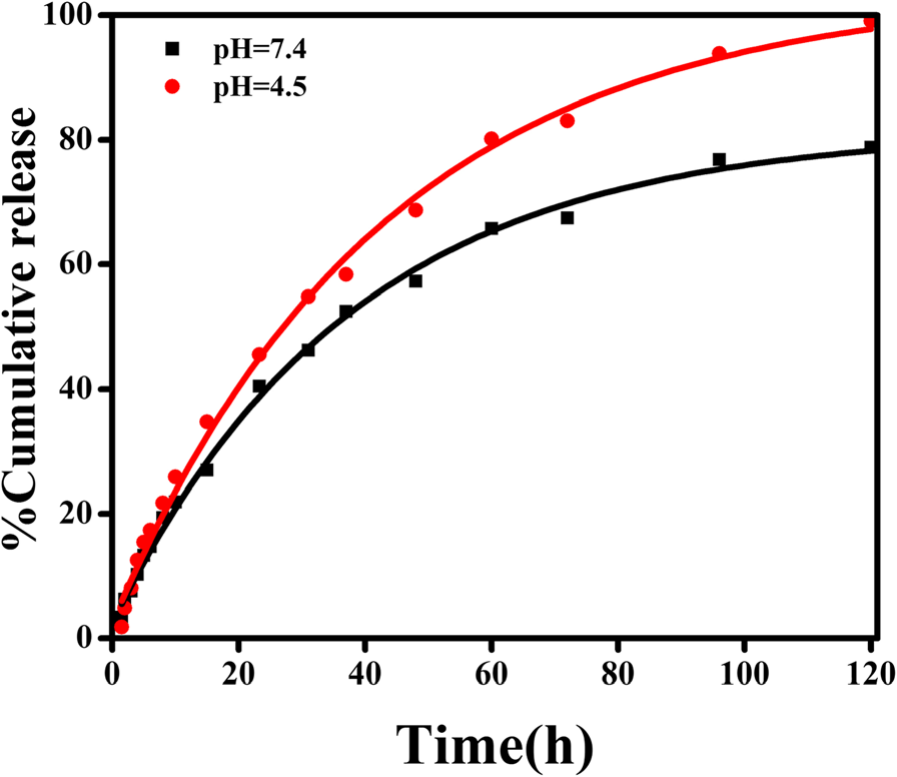
Release profiles of etoposide from ECCNBs under simulated physiological conditions (pH 7.4 and pH 4.5 at 37 °C)

Figure 2 shows the drug release kinetics of VP16 from ECCNBs. The drug release behavior from ECCNBs was examined under the two pH values which simulate the cellular exterior (pH 7.4) and intracellular lysosome (pH 4.5) [32, 33], respectively. During the first 24 h, the speed of release was fast, which may be attributed to the physical adsorption of drugs. After that point, a sustained release from ECCNBs could be observed. Compared to the amount release which was approximately 78 % at pH 7.4, the cumulative drug release is up to 98 % at pH 4.5 in 120 h. If oral administration is chosen, the ECCNBs can ensure a stable delivery of etoposide during blood circulation. That is to say, on the one hand, CCNBs could minimize the drug loss in the blood where the pH value is neutral and selectively release drugs when they are internalized into the target cells. On the other hand, most of the biodegradable ECCNBs can decompose in the vicinity of the tumor endothelium through the EPR effect. A stable and fast VP16 release could be triggered in response to the microenvironment of tumor tissues. In terms of the delivery efficiency, ECCNBs would be more promising than native VP16.

**Fig. 3:**
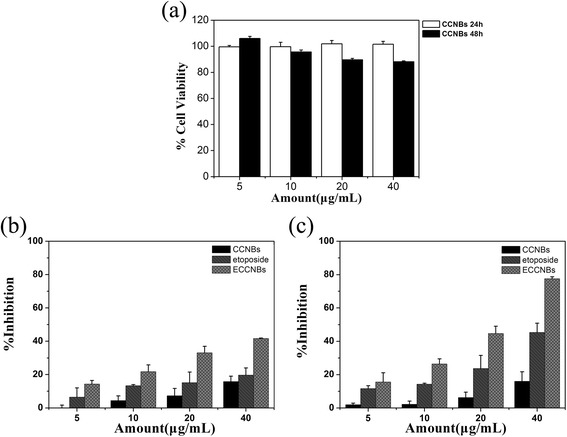
Growth inhibition assay results for 293T cell line with CCNBs after 24 and 48 h incubation (**a**). Growth inhibition assay results for SGC-7901 cell line with CCNBs, free etoposide, and ECCNBs after 24 and 48 h incubation (**b**, **c**). Diagrams were plotted as etoposide concentrations of 5, 10, 20, and 40 μg/mL, respectively. All experiments were carried out in triplicate

The data (Fig. 3a) clearly showed that the carrier (CCNBs) did not show any cytotoxicity (average residual remained over 90 %) against normal cells (293T cells). It demonstrates a good biocompatibility. Figure 3b, c shows the inhibition effect of CCNBs, VP16, and ECCNBs, against SGC-7901 cell growth. The figure demonstrates that the suppression of SGC-7901 cell growth was time and concentration dependent. The inhibition rates of the free VP16 and ECCNBs are 45.37 and 77.53 % at 48 h, respectively. Clearly, it shows that ECCNBs have a higher suppression efficiency compared to the native VP16. It is likely that the good dispersivity of ECCNBs leads to a greater cellular uptake.

**Fig. 4:**
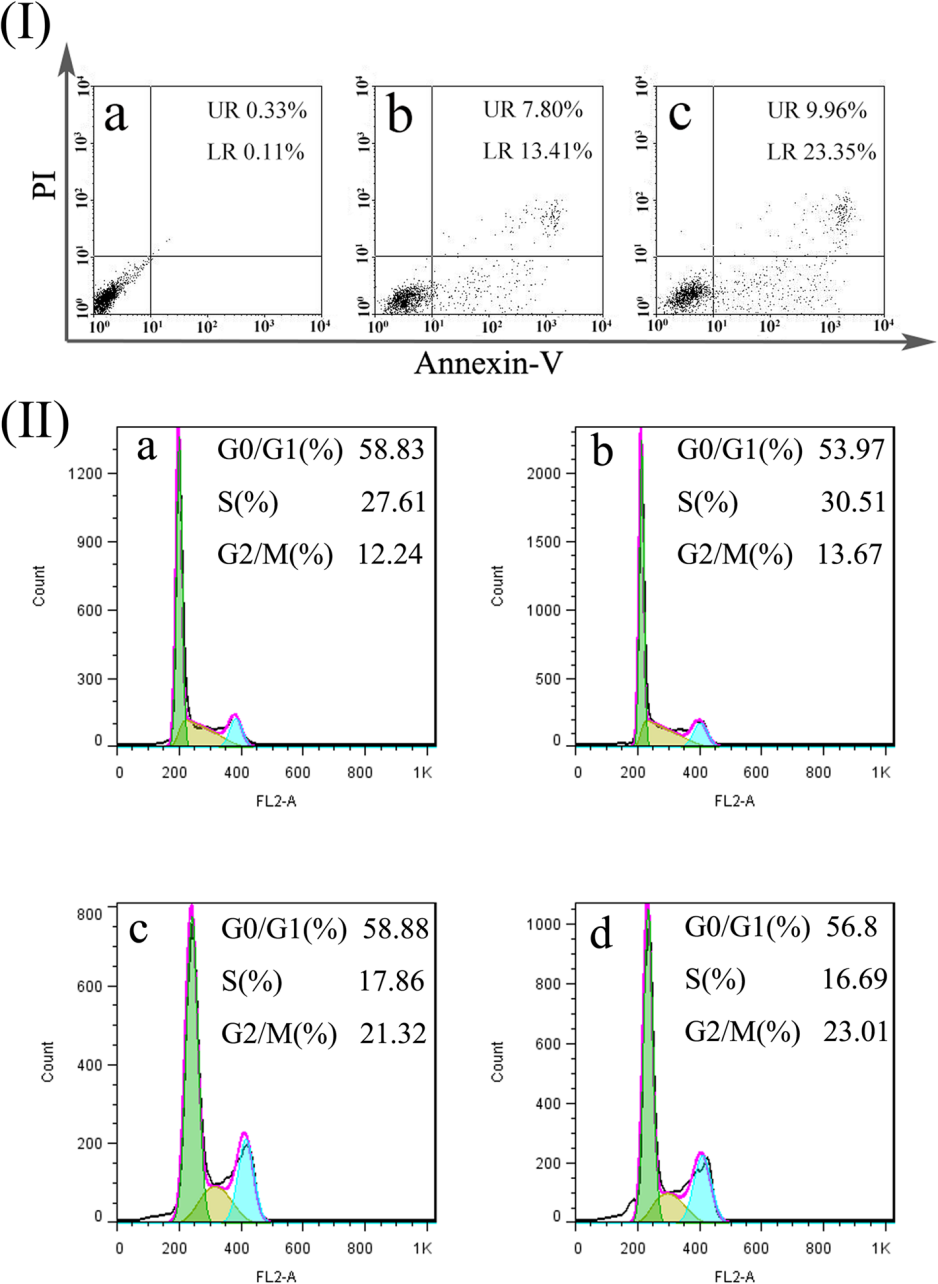
*I* FACS analysis of SGC-7901 cells stained with AnnexinV-FITC and PI. Cells did not treat with any agents as blank control (***a***), cells apoptosis induced by etoposide (***b***), cells treated with the ECCNBs (***c***). In all panels, LR represents early apoptosis and UR represents late apoptosis. *II* Analysis of cell cycle distribution by flow cytometry: SGC-7901 cells set as control group (***a***); SGC-7901 cells treated with CCNBs (***b***); SGC-7901 cells treated with etoposide (*c*) (30 μg/mL); SGC-7901 cells treated with ECCNBs (***d***)

The mechanism of cell growth inhibition was studied by using Annexin V-FITC Apoptosis Detection Kit. Figure 4(I) illustrates the percentages of early and late apoptotic cells of the apoptosis assay, which stained cells with AnnexinV-FITC and PI labeling. Early apoptosis was characterized by plasma membrane reorganization, which was characterized by positive staining for Annexin V-FITC. Later stage apoptosis was characterized by DNA damage, which was detected by positive staining for both Annexin V and PI.

We treated with ECCNBs (20 μg/mL) the free etoposide against SGC-7901 cells for 24 h prior to FACS analysis. Moreover, we set cells without any addition as control. SGC-7901 cells without any additive showed 0.11 % early apoptosis and 0.33 % later apoptosis in Fig. 4(Ia). The treatment with etoposide led to 13.41 % early apoptosis and 7.80 % later apoptosis (Fig. 4(Ib)). Observably, the percentage of cells treated with ECCNBs that reached later apoptosis increased to 9.96 % and early apoptosis increased to 23.35 % (Fig. 4(Ic)).

It is well known that topoisomerase II of VP16-induced cleavage of DNA can mediate the formation of chromosomal translocation breakpoints, which can lead to and is responsible for the expression of oncogenic factors [12]. VP16 can cause apoptosis cascade by coupling DNA damage to p53 phosphorylation through the action of DNA-dependent protein kinase in gastric cancer cells [34].

Compared with free VP16 and untreated controls, the percentage of both early apoptosis and later apoptosis in ECCNBs group is significantly increased. It demonstrates that ECCNBs were able to induce the apoptosis processes among gastric cancer cells and readily caused the cells to die. The results show that ECCNBs can enhance the efficiency of anti-tumor effect.

**Fig. 5:**
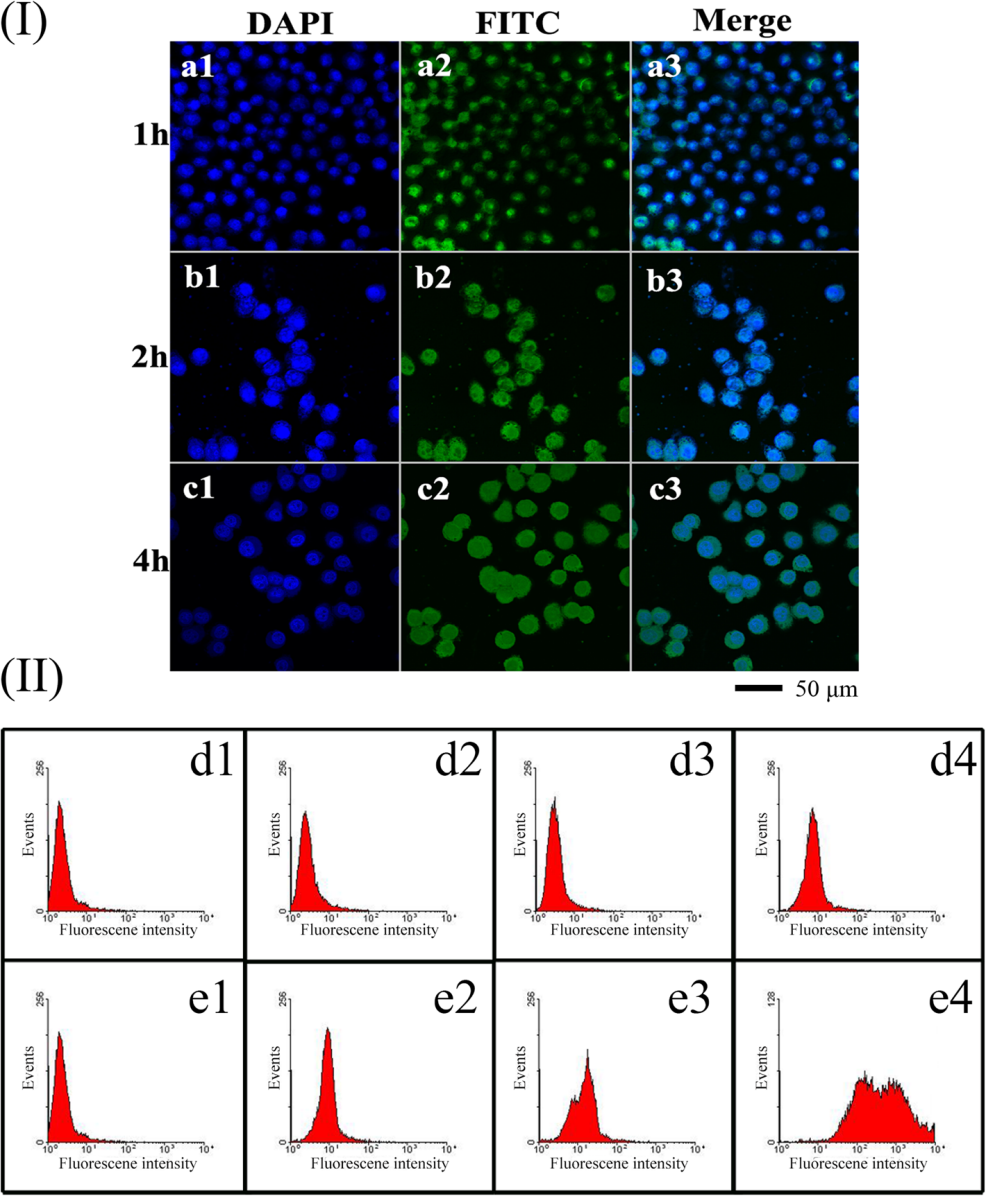
*I* Confocal laser scanning microscopy (CLSM) images of the ECCNBs (*a*–*c*) on SGC-7901 cells at the predetermined point of 1, 2, and 4 h, respectively. In each case, 1, 2, and 3 indicate DAPI, FITC, and Merge, respectively. *II* SGC-7901 cells were treated with 30 μg/mL etoposide in two forms of ECCNBs (*e2*–*e4*) and void etoposide (*d2*–*d4*). As the plots show, the number of events (*y*-axis) with high fluorescence intensity (*x*-axis) increases by 4 h incubation with ECCNBs but without any evident change for void etoposide. Negative control (*d1*, *e1*) includes non-treated cells to set their auto-fluorescence as “0” value

We evaluated the high therapeutic effect of ECCNBs by the uptake behavior of SGC-7901 cells. As shown in Fig. 5(I), ECCNBs passed through the cytomembrane and eventually assembled in the nucleus at 1, 2, and 4 h, respectively. The results show the time-dependent cellular uptake. CLSM images show that the CCNB carriers could aggregate around the nucleus and even directly intrude into the nucleus. The enhanced intracellular delivery effective therapy may result from the pH-sensitive release.

Kinetic assessment of void etoposide (Fig. 5(d2, d3, d4)) and ECCNBs (Fig. 5(e2, e3, e4)) uptake was showed by plotting the fluorescence peak at different incubation time of 1 h (d2, e2), 2 h (d3, e3), and 4 h (d4, e4). The number of events with the high intensity of 30 μg/mL etoposide increased with its uptake into cells about 4 h of incubation time. As shown in Fig. 5(II), FACS analysis showed the cells treated with ECCNBs showed better profound fluorescence intensity. It indicates that CCNBs internalized have a better efficiency than native etoposide. The result of fluorescence intensity of the ECCNBs is in agreement with the result of CLSM images.

We examined the effect of etoposide and ECCNBs on SGC-7901 cell cycle by analyzing the DNA content of cells, which stained with propidium iodide by flow cytometry. After cultured with 30 μg/mL etoposide and ECCNBs for 24 h, the cell cycles were conducted by flow cytometry. As demonstrated in Fig. 4(II), the G_2_/M cell cycle arrest was significantly increased after etoposide and ECCNBs treatment in SGC-7901 cells. Based on these results, we believe that etoposide might induce G_2_/M arrest and exert through its toxic effects on G_2_ phase cells and inhibit G_2_/M transition of SGC-7901 cells. Further studies are needed to deepen the insight because the underlying mechanism is not known.

**Fig. 6:**
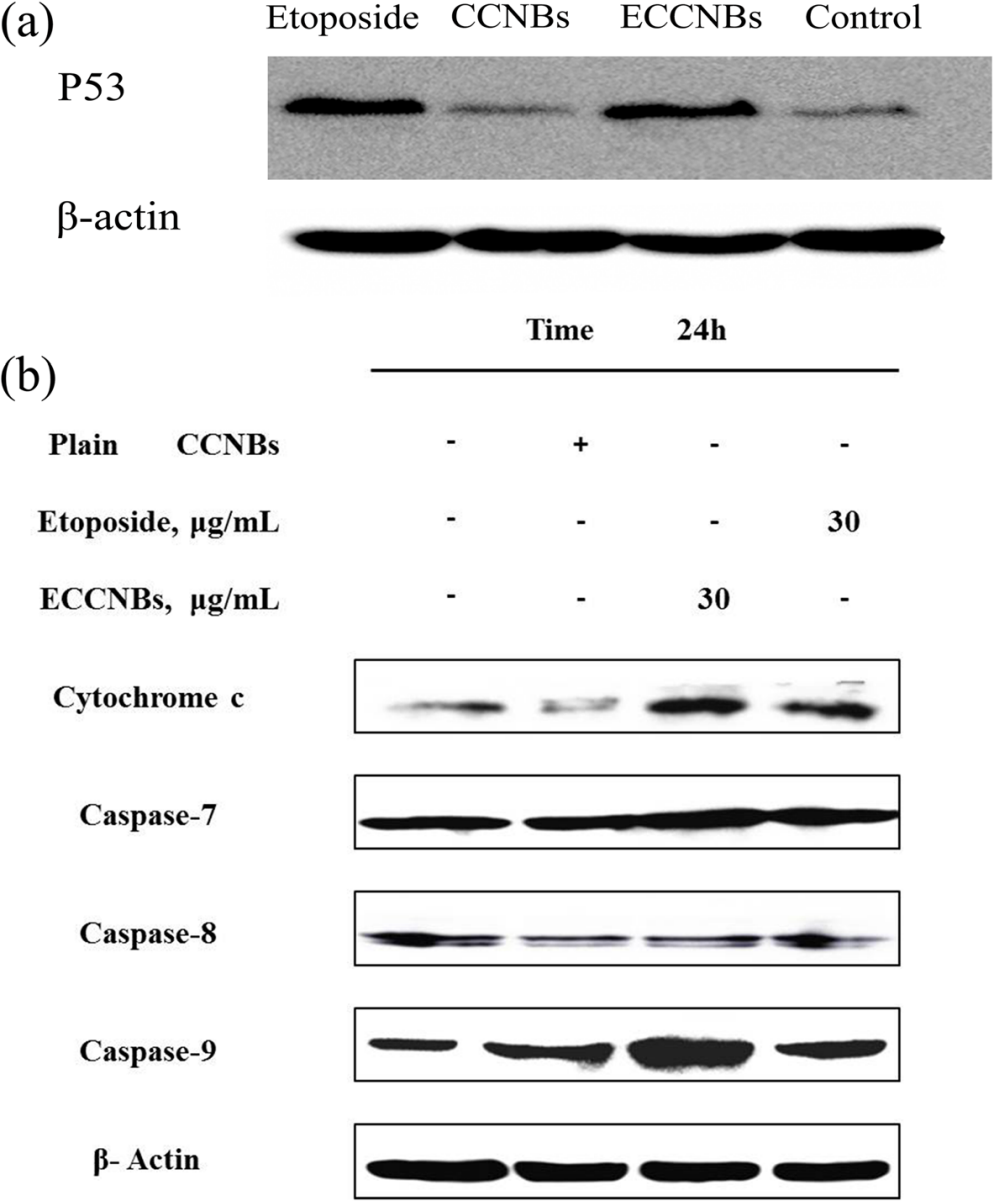
**a** Comparative effects of native etoposide, CCNBs, and ECCNBs on p53-related signaling pathway in SGC-7901 cells by immunoblotting with specific antibodies mentioned in Materials and Methods. Effects of native etoposide, CCNBs, and ECCNBs on the expression of p53. **b** Western blot illustrating expression of apoptotic markers in SGC-7901 cells. Cells in culture were exposed to 30 μg/mL of native etoposide, CCNBs, and ECCNBs for indicated time periods. Effects of native etoposide, CCNBs and ECCNBs on the expression of caspase-7, caspase-8, caspase-9, and Cyt C

As shown in Fig. 6a, our western blot results showed an increase in the expression of p53 (band at 53 kDa) of etoposide and ECCNB-treated SGC-7901 cells compared to the positive control. Wild-type p53 (a crucial G_1_ checkpoint) is well known to induce either cell cycle arrest or apoptosis in cells that undergo DNA damage [35]. It demonstrated that ECCNBs may induce G_2_/M cell cycle arrest and cell apoptosis possible through a p53-related pathway when exposed on SGC-7901 cells from this study.

The apoptosis pathway can be divided into caspase-independent and caspase-dependent branches in mammalian cells. The activation of caspase family can lead to the apoptosis of most of the proteolytic cleavages [36]. Caspases (a family of cysteine acid proteases) are cleaved following proteolytic activation in early apoptosis [37]. The expression of caspase family is one of the commonly diagnostic tools for the detection of apoptosis activity. Some of the caspase family proteins such as caspase-7, caspase-8, and caspase-9 are widely accepted to mediate the apoptotic pathway. In this regard, western blot analysis was consistent to the apoptosis result and confirmed our assumption that the expression of caspase-9 and Cyt C was increased by ECCNBs in SGC-7901 cell.

p53 mainly induced Bax and restrain the Bcl-2, which can lead to the release of ATP and Cyt C in the cytoplasm. ATP and Cyt C can combine with Apaf-1 to activate caspase-9 [38]. p53 also can promote related gene expression of oxidative stress. Expression product can produce reactive oxygen species. The reactive oxygen species can trigger the release of Cyt C, and apoptosis initiation activated caspase protein which leads to apoptosis [39]. As shown in Fig. 6b, the western blot results showed an increase in the expression of Cyt C and caspase-9 of ECCNB-treated SGC-7901 cells compared to that of etoposide. It suggests that ECCNBs can present an increased apoptotic effect through mitochondrial pathway than free etoposide.

## Conclusions

In this study, we developed a facile method to prepare CCNBs with citric acid as a crystal modifier to deliver VP16 for cancer therapy. The experiments on CCNBs as carriers for VP16 demonstrated the controlled release profile and enhanced cytotoxicity by increasing cellular uptake and apoptosis against tumor cell. The experiments of cell cytotoxicity and cell apoptosis demonstrated that ECCNBs were more efficient than native VP16 in delivery activity. ECCNBs can present an increased apoptotic effect through mitochondrial pathway than free VP16. It can be foreseen that CCNBs is a promising drug carrier to store the anti-cancer drug VP16 for cancer therapy.

## Additional file

Additional file 1: Figure S1. FTIR spectra of CCNBs (a) ECCNBs (b) and etoposide (c).
