# Supplementary figures and images for: Synthesis of CaCO3 Nanobelts for Drug Delivery in Cancer Therapy
Source: Nanoscale Res Lett. 2015 May 27;10:239. doi: 10.1186/s11671-015-0948-6 (PMC4456597; doi:10.1186/s11671-015-0948-6)

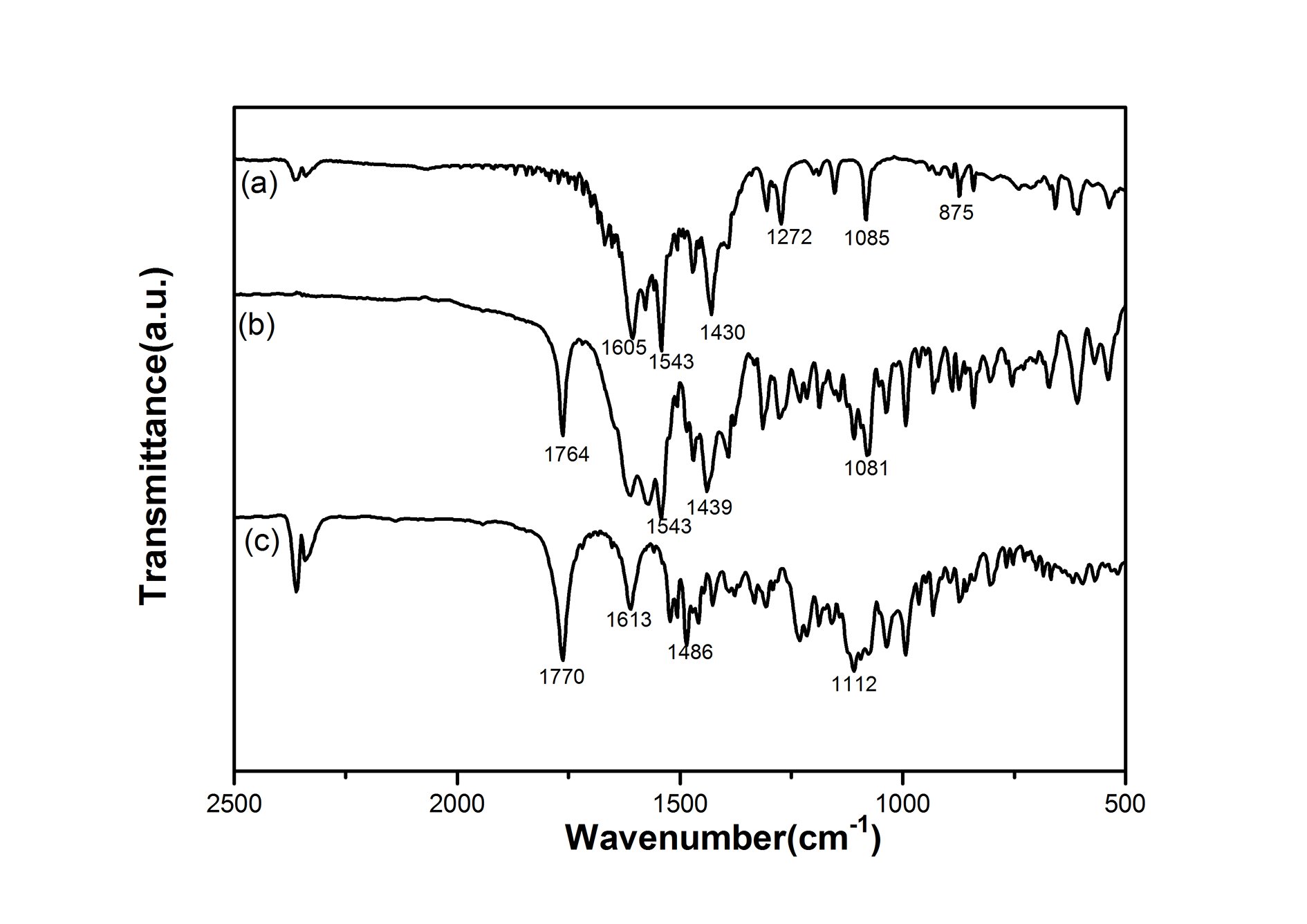

Supplement: Additional file 1: Figure S1. — FTIR spectra of CCNBs (a) ECCNBs (b) and etoposide (c). [file 11671_2015_948_MOESM1_ESM.tiff]
